# Draft Genome Sequence of *Cytophagales* sp. Strain WSM2-2, Isolated from Garden Soil

**DOI:** 10.1128/MRA.01238-20

**Published:** 2021-01-14

**Authors:** Masataka Aoki, Mamoru Oshiki, Mizuki Iwagaki, Masashi Hatamoto, Takashi Yamaguchi, Nobuo Araki

**Affiliations:** a Department of Civil Engineering, National Institute of Technology, Wakayama College, Gobo, Wakayama, Japan; b Division of Environmental Engineering, Faculty of Engineering, Hokkaido University, Sapporo, Hokkaido, Japan; c Department of Civil Engineering, National Institute of Technology, Nagaoka College, Nagaoka, Niigata, Japan; d Department of Civil and Environmental Engineering, Nagaoka University of Technology, Nagaoka, Niigata, Japan; e Department of Science of Technology Innovation, Nagaoka University of Technology, Nagaoka, Niigata, Japan; University of Southern California

## Abstract

We report the draft genome sequence of *Cytophagales* sp. strain WSM2-2, isolated from garden soil. A 5.5-Mb genome sequence comprising four contigs was successfully obtained using Illumina NovaSeq and MinION sequencers. This draft genome sequence will contribute to the genomic knowledge of the bacterial order *Cytophagales.*

## Abstract (precis)

We report the draft genome sequence of the *Cytophagales* sp. strain WSM2-2, isolated from garden soil. A 5.5-Mb genome sequence comprising four contigs was successfully obtained using Illumina NovaSeq and MinION sequencers. This draft genome sequence will contribute to the genomic knowledge of the bacterial order *Cytophagales.*

## ANNOUNCEMENT

*Bacteroidetes* bacteria colonize different ecological niches, including soil, oceans, and freshwater (1), and play significant roles in Earth’s elemental cycles. Many cultivated representatives belonging to the order *Cytophagales* within the phylum *Bacteroidetes* degrade several biomacromolecules (2). However, their metabolic capabilities and ecological roles remain unclear due to the limited availability of cultivated representatives and sequenced genomes.

Here, we report the draft genome of *Cytophagales* sp. strain WSM2-2, isolated from garden soil located in Shirahama, Wakayama, Japan, using the filtration-acclimatization method (3). In brief, ∼5 g of garden soil (0- to 10-cm depth from the surface) suspended in phosphate-buffered saline (pH 7.5) was filtered through three serially connected filters (0.22 μm). Further, 20 ml of the filtrate was mixed with 20 ml of R2A broth (4) and then statically incubated in the dark at 20°C for 2 months. The turbid culture was subsequently spread onto a half-strength R2A agar plate (15 g/liter of agar) and incubated in the dark at 20°C for 2 weeks. Strain WSM2-2 was obtained by picking a single colony from the agar plate.

Prior to sequencing, strain WSM2-2 was cultivated statically in R2A broth at 25°C for 1 month, and genomic DNA was extracted using a DNeasy blood and tissue kit (Qiagen). The same genomic DNA extract was used for Illumina and Nanopore sequencing. For Illumina sequencing, the genomic DNA was sheared into 350-bp fragments using an E200 ultrasonicator (Covaris). No DNA size selection or fragmentation was performed for Nanopore sequencing. Genomic DNA was sequenced using 150-bp paired-end Illumina sequencing using NovaSeq (TruSeq DNA PCR-free library prep kit) and Nanopore sequencing using MinION (flow cell vR9 and rapid sequencing kit; Oxford Nanopore Technologies [ONT]). Default parameters were set for all software used, unless specified otherwise. The fast5 files generated by the Nanopore sequencing were base-called using Guppy v2.3.5 (ONT). Only the Nanopore sequence reads stored in the “pass” folder were used in the following steps. The Illumina and Nanopore sequencing produced 27,085,198 and 57,294 (*N*_50_ length, 7,455 bp) raw sequence reads, respectively. Illumina sequence reads were trimmed and filtered using Trimmomatic v0.36 (Phred quality score cutoff of 30) (5). Nanopore sequence reads were trimmed and filtered using Nanofilt v2.3.0 (6) using a minimum average read quality score of 7, minimum read length of 500 bp, and trimming of 50 bp from the 5′ end. Hybrid assembly of sequence reads was performed using Unicycler v0.4.1 (7). Gene prediction and annotation were performed using the DFAST pipeline v1.2.4 (8).

The genome of WSM2-2 comprised four contigs (>500 bp) with a total length of 5,547,679 bp (*N*_50_ length, 5,512,392 bp). The genome coverage was 242×, and the G+C content was 43.6%. The CheckM (9) estimation revealed that the completeness of the genome was 100.0%, with 0.0% contamination. In total, 4,786 coding sequences, 6 rRNA genes, and 47 tRNA genes were identified. Nucleotide BLAST (10) analysis of the 16S rRNA gene sequence identified Ohtaekwangia koreensis 3B-2^T^ (89.98% sequence identity) (11) and Chryseolinea soli KIS68-18^T^ (89.81% sequence identity) (12) as the closest validated species.

### Data availability.

The draft genome sequences have been deposited in DDBJ/ENA/GenBank under the accession numbers BNHL01000001, BNHL01000002, BNHL01000003, and BNHL01000004. The Nanopore and Illumina read data can be found in the Sequence Read Archive under the accession numbers DRX238110 and DRX238111, respectively.
